# Genome-Wide Association Study for Drought Tolerance in Cowpea (*Vigna unguiculata* (L.) Walp.) at Seedling Stage Using a Whole Genome Resequencing Approach

**DOI:** 10.3390/ijms26125478

**Published:** 2025-06-07

**Authors:** Waltram Ravelombola, Haizheng Xiong, Gehendra Bhattarai, Aurora Manley, John Cason, Hanh Pham, Bazgha Zia, Beiquan Mou, Ainong Shi

**Affiliations:** 1 Texas A&M AgriLife Research, 11708 Highway 70 South, Vernon, TX 76384, USA; 2 Department of Soil and Crop Sciences, Texas A&M University, 370 Olsen Blvd., College Station, TX 77843, USA; 3 Department of Horticulture, 316 Plant Sciences Building (PTSC), University of Arkansas, Fayetteville, AR 72701, USA; 4 Texas A&M AgriLife Research, 1129 North US Highway 281, Stephenville, TX 76401, USA; 5 Texas A&M AgriLife Research, 1102 East Drew Street, Lubbock, TX 79403, USA; 6 United States Vegetable Lab (USVL), 2700 Savannah Hwy, Charleston, SC 29414, USA; 7 Sam Farr United States Crop Improvement and Protection Research Center, 1636 E. Alisal Street, Salinas, CA 93905, USA; beiquan.mou@usda.gov

**Keywords:** cowpea, genetics, drought

## Abstract

Despite the fact that cowpea is one of the most drought-tolerant legumes, some genotypes with a high yield under well-watered conditions have been shown to be susceptible to drought stress, thus requiring further improvement. The objectives of this study were to conduct a genome-wide association study (GWAS) for drought tolerance in cowpea. A total of 331 cowpea genotypes were evaluated for drought tolerance. After SNP filtering, 5,884,299 SNPs were used to conduct GWAS using BLINK. The results showed: (1) a significant GWAS peak defined by a cluster of 196 significant SNPs and mapped on a 210 kb region of chromosome 5 was identified to be a good locus candidate for tolerance to trifoliate leaf chlorosis under drought stress in cowpea, (2) a strong GWAS peak was found towards the end of chromosome 1 and this peak was a good candidate locus for tolerance to unifoliate leaf chlorosis under drought stress in cowpea, and (3) a total of 25 SNPs located on chromosomes 1, 3, 5, and 11 were significantly associated with plant greenness under drought stress. This study provides a better understanding of the molecular genetics of drought tolerance in cowpea and the findings can be expanded to other crop species.

## 1. Introduction

Breeding programs aiming at developing and releasing cultivars having the ability to better withstand drought conditions have been of interest over the last decades since the randomness of rainfall unfavorably impacts crop production. Severe drought conditions have been reported to lead to significant crop yield losses and plant death [1,2]. Drought-related issues are growing threats impairing legume production in tropical and sub-tropical areas [3]. Cowpea [*Vigna unguiculata* (L.) Walp.] is one of the most widely grown legumes in these regions [4].

Cowpea, (2n = 2x = 22), is a legume consumed for its protein. It belongs to the family *Fabaceae* [5]. Previous investigations have shown that cowpea originated from Africa [6]. In regions where cowpea is widely grown, limited access to water undermines cowpea production [7]. Cowpea cultivation is rain-dependent, and scarcity of water occurring at early vegetative growth is detrimental to cowpea production in spite of its high degree of drought tolerance over other crops [8]. Therefore, improving the drought tolerance of existing cowpea cultivars could address the increasing constraints imposed by drought conditions. In addition, with a relatively small genome size estimated to be 620 Mb [9] and a better ability to withstand drought [10,11], cowpea has been considered as a model crop for understanding the drought mechanism in other crops [3].

A QTL for drought tolerance at seedling stage in 128 cowpea RILs derived from the cross between IT93K503-1 (drought-tolerant) and CB46 (drought-susceptible) was previously reported [12]. A total of 306 amplified fragment length polymorphism (AFLP) markers were used. The results revealed 10 drought-related QTLs based on recovery dry weight, visual rating of stem greenness, and leaf senescence, and percent leaf damage under both greenhouse and field conditions. Another study suggested homology between seven previously reported drought QTLs and drought-related or abiotic stress-induced expressed sequence tags (ESTs) derived from cowpea or other plants [13]. Since the previous number of QTLs was significantly large and the QTL resolution (22.7 cM to 76.6 cM) was poor, using such results for breeding purposes might be challenging [12].

Efforts toward effectively developing and improving crop drought-tolerant cultivars require knowledge pertaining to the genetics underlying such traits. Sequencing technologies have been tremendously improved recently, allowing scientists to perform whole genome (re)sequencing of crops for a reasonable cost even if only a reference genome is partially available. Further, the gaps existing between model and crop species have been progressively filled over the last few years [14], which will speed up the discovery of genes controlling traits of agronomic interests. Whole genome (re)sequencing permits the discovery of a large set of SNPs which can be used for genome-wide association studies (GWAS) [15,16]. In regard to drought-related studies involving GWAS, previous reports have been proven to be promising at identifying molecular markers or regions of the genome associated with tolerance to drought. A total of 223 barley (*Hordeum vulgare* L.) accessions were evaluated for drought and a GWAS was conducted using 710 Dart markers, 61 SNPs, and 45 SSRs [17]. In soybean [*Glycine max* (L.) Merr.], the carbon isotope ratio (δ^13^C) was used as a surrogate for assessing water use efficiency in a soybean panel consisting of 373 genotypes [18]. A total of 12,347 SNPs were used for GWAS and results showed that 39 SNPs were significantly associated with δ^13^C. In model plants such as *Arabidopsis*, a total of 324 natural accessions of *Arabidopsis* were evaluated for drought tolerance, and six time-dependent QTLs for that trait were reported [19]. In rice (*Oryza sativa* L.), a total of 10 previously reported genes for drought tolerance were found using the GWAS approach [20]. A total of 175 rice accessions were analyzed and GWAS involved 150,325 SNPs for that study. A total of 140 canola (*Brassica napus* L.) accessions were phenotyped for drought tolerance and GWAS allowed the identification of 16 loci associated with drought tolerance [21]. Candidate genes for glutamate-cysteine ligase and aldehyde dehydrogenase associated with stomata density under drought conditions were identified in *Medicago Truncatula* Gaertn. through GWAS [22]. In regard to common bean (*Phaseolus vulgaris* L.), traits consisting of wilting and leaf growth rate under drought conditions were evaluated in a panel of 96 genotypes, and GWAS revealed 27 significant SNPs associated with drought tolerance [23]. A marker–trait association involving 201 maize (*Zea mays* L.) inbred lines using 41,101 SNPs was conducted, and the results revealed 206 SNPs associated with drought tolerance-related traits with 115 candidate genes [24].

QTL mapping in biparental crossings has also been used to identify the genetic regions associated with drought tolerance. However, few genes have been identified from previously identified QTLs [25]. GWAS, a linkage disequilibrium-based approach, provides greater resolution, thus reliably allowing the identification of specific regions in the genome associated with traits [26]. GWAS might be affected by population stratification, which can be addressed by conducting a population structure analysis [27,28,29,30,31,32]. The use of SNPs as molecular markers has been shown to be rewarding in the field of plant breeding [33]. To our knowledge, reports on GWAS for drought tolerance in cowpea remain limited despite the power of this technology in identifying genomic regions associated with traits of interest in agriculture and the potential of cowpea to be used as a model crop for studying drought tolerance mechanism in plants [32]. This study aimed to conduct a genome-wide analysis study (GWAS) for drought tolerance at seedling stage in cowpea, and to identify the SNP markers and candidate genes for drought tolerance using a whole-genome resequencing approach.

## 2. Results

### 2.1. Population Structure and Genetic Diversity

**Figure 1:**
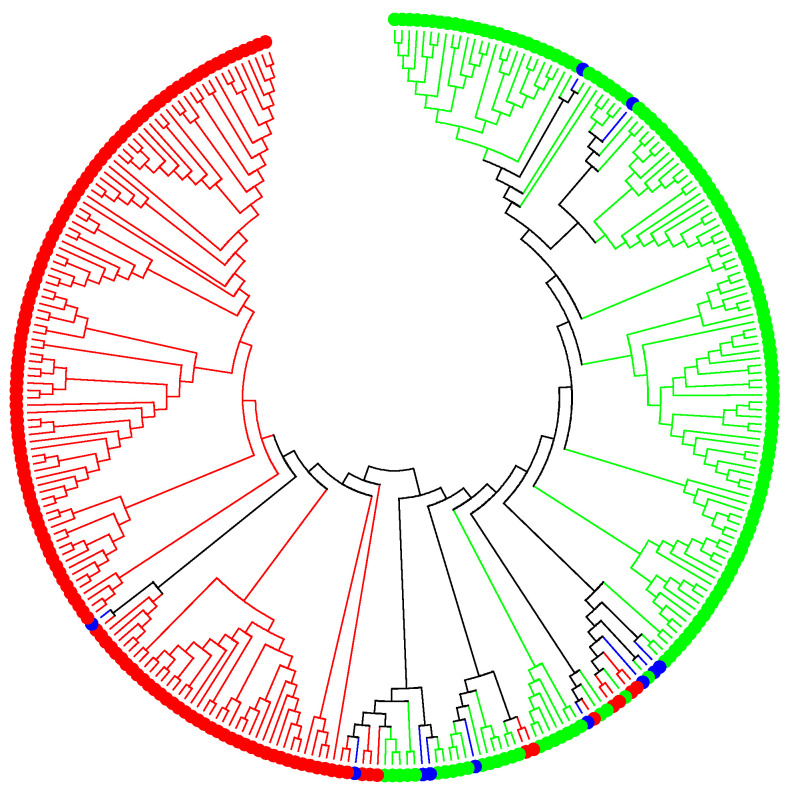
Population structure and genetic diversity analysis.

The population structure and genetic diversity analysis showed that this population can be divided into two subpopulations. Figure 1 shows a combined genetic diversity and population structure analysis. The green dots represent subpopulation 1 (52%), whereas the red dots are subpopulation 2 (44%). This population has a low level of admixture, shown in blue dots (4%). The average SNP density per chromosome was 111 bp, 94 bp, 108 bp, 47 bp, 112 bp, 81 bp, 88 bp, 94 bp, 112 bp, 49 bp, and 59 bp for chromosomes 1, 2, 3, 4, 5, 6, 7, 8, 9, 10, and 11, respectively.

### 2.2. First Trifoliate Leaf Chlorosis Under Drought Stress

**Figure 2:**
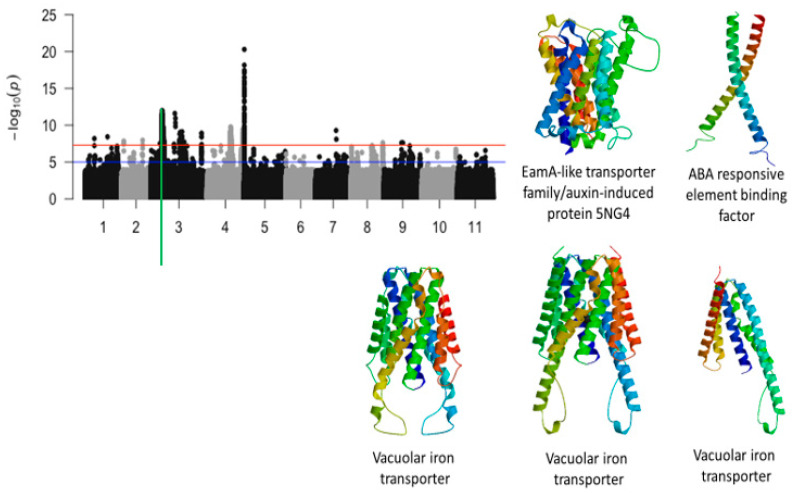
Manhattan plot for chlorosis trifoliate leaf tolerance in cowpea where the vertical green line defines a genomic region harboring genes encoding for EamA-like transporter/auxin-induced protein 5NG4, ABA responsive element binding factor, and vacuolar iron transporters.

**Figure 3:**
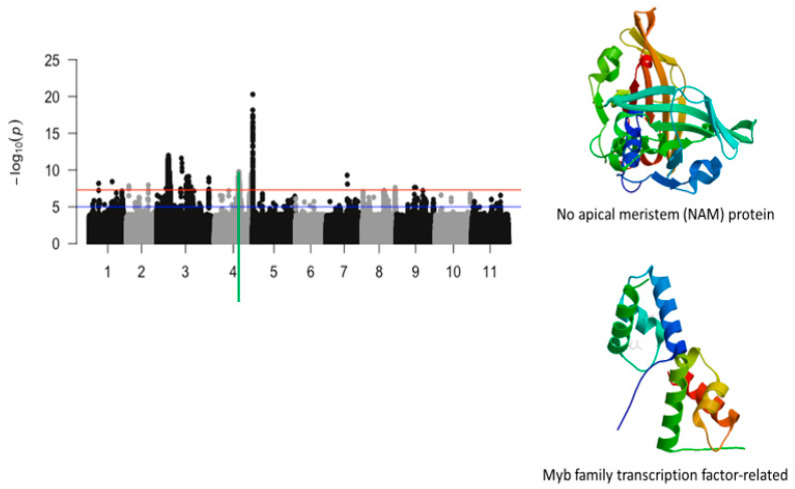
Manhattan plot for chlorosis trifoliate leaf tolerance in cowpea where the vertical green line defines a genomic region harboring genes encoding for no apical meristem (NAM) protein and Myb family transcription factor-related.

**Figure 4:**
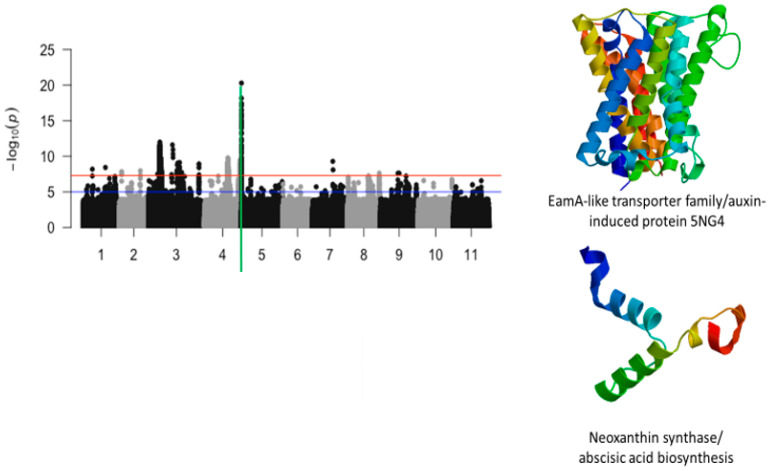
Manhattan plot for chlorosis trifoliate leaf tolerance in cowpea where the vertical green line defines a genomic region harboring genes encoding for EamA-lie transporter family/auxin-induced protein 5NG4 and neoxanthin synthase/abscisic acid biosynthesis.

Of the 5,884,299 SNPs used to conduct GWAS for tolerance to first trifoliate leaf chlorosis under drought stress in cowpea, a total of 1047 SNPs were above the threshold (Table 1) (Figure 2, Figure 3 and Figure 4). Significant SNPs were located on chromosomes 1, 2, 3, 4, 5, 7, and 9. The number of significant SNPs was 2, 2, 1232, 610, 196, 2, 1, and 2 for the chromosomes 1, 2, 3, 4, 7, and 9, respectively. LOD values (−log_10_(*p*-value)) for the significant SNPs varied from 7.52 to 20.29. One of the most interesting findings from this study was the identification of four significant loci associated with tolerance to first trifoliate leaf chlorosis under drought stress. These loci were mapped at the start of chromosome 3, in the middle of chromosome 4, towards the end of chromosome 4, and at the beginning of chromosome 5.

The significant locus found on a 1.3-Mb region of chromosome 3 was defined by a total of 1149 SNPs. Functional annotations of the candidate genes found within regions showed proteins that were involved in hormone-induced responses such as auxin and abscisic acid. This genomic region was also characterized by a significant cluster of biomolecule transporters (Figure 2). Tertiary structure analysis of the proteins that were derived for the candidate genes are shown in Figure 2. For example, a cluster of vacuolar iron transporters were mapped on a 30 kb genomic region and proteins derived from these transporters were slightly different from each other (Figure 2). The SNPs that were found within or in the vicinity of these vacuolar iron transporters were Vu03_13295491, Vu03_13297714, and Vu03_13302250 (Table 1). The candidate genes associated with the vacuolar iron transporters were *Vign03g135700.1*, *Vign03g135800.1*, and *Vign03g135900.1* (Table 1). The SNP that was found within the annotated gene associated with EamA-like transporter family/auxin-induced protein 5NG4, *Vigun03g136600.1*, was Vu03_13382599 (LOD = 9.59). In addition, an annotated gene, *Vigun03g137500.1*, encoding for an ABA responsive element binding was found in the vicinity of Vu03_13509429 (LOD = 10.25). Tolerance to trifoliate leaf chlorosis was assessed based on the level of leaf greenness. As expected, results identified a significant SNP, *Vu03_14815803* (LOD = 8.79), that was found on chromosome 3 and located within an annotated gene encoding for a chlorophyll a/b binding protein. In addition, a significant SNP, Vu03_36340055, was also mapped in the vicinity of an annotated gene encoding for ABC-2 type transporter family protein (Table 1). Other genomic regions of chromosome 3 also harbored significant SNPs associated with tolerance to trifoliate leaf chlorosis under drought stress in cowpea. However, these regions were less gene-dense, and the annotated genes found within these regions had functional annotations that were less relevant to plant abiotic stress.

Chromosome 4 had two significant loci defined by about 800 kb and 100 bk genomic regions, respectively (Figure 3). The 800 bk genomic region harbored a total of 484 significant SNPs and the second one had 69 SNPs. Of these SNPs, 19 were mapped within the structure of annotated genes that had functional annotations relevant to plant abiotic stress. These SNPs consisted of Vu04_26966450 (LOD = 8.37), Vu04_27157237 (LOD = 8.21), Vu04_27241963 (LOD = 8.3), Vu04_27298716 (LOD = 8.22), Vu04_27342140 (LOD = 8.56), Vu04_27505387 (LOD = 8.51), Vu04_27528973 (LOD = 8.1), Vu04_27714135 (LOD = 8.72), Vu04_27716250 (LOD = 8.35), Vu04_27778870 (LOD = 7.67), Vu04_27786623 (LOD = 9.08), Vu04_27797389 (LOD = 8.37), Vu04_27830859 (LOD = 7.81), Vu04_27913211 (LOD = 7.8), Vu04_27913980 (LOD = 8.06), Vu04_41785910 (LOD = 8.5), Vu04_41800041 (LOD = 7.67), Vu04_41826262 (LOD = 8.11), and Vu04_41832927 (LOD = 8.09) (Table 1). Two annotated genes, *Vigun04g110600.1* and *Vigun04g110800.1*, having functional annotations that were directly relevant were found within the 800 kb locus associated with tolerance trifoliate leaf chlorosis. *Vigun04g110600.1* and *Vigun04g110800* encodes for no apical meristem protein (NAM) and a Myb-family protein. Structural analysis of these two proteins was investigated and is visualized in Figure 3.

The most significant finding was the identification of a strong locus associated with tolerance to first trifoliate chlorosis on chromosome 5 (Figure 4). The locus was defined by a 210 kb region and harbored a total of 196 significant SNPs. In this region, LOD (−log_10_(*p*-value)) values varied from 7.52 to 20.29. SNPs with the highest LOD values were Vu05_539746 (LOD = 17.28), Vu05_539750 (LOD = 17.07), Vu05_539753 (LOD = 17.45), Vu05_539879 (LOD = 16.48), Vu05_539880 (LOD = 16.48), Vu05_539926 (LOD = 16.52), Vu05_540522 (LOD = 18.16), Vu05_540561 (LOD = 20.29), Vu05_541044 (LOD = 16.5), Vu05_541198 (LOD = 17.4), and Vu05_548993 (LOD = 17.18). Two SNPs, Vu05_540561 (LOD = 20.29) and Vu05_560665 (LOD = 14.25), were located within the structure of *Vigun05g006300.1* and *Vigun05g006500.1*, respectively. These annotated genes encode for an auxin-induced protein and a neoxanthin synthase involved in the abscisic acid biosynthesis. Chromosomes 7 and 8 also harbored significant SNPs associated with tolerance to trifoliate leaf chlorosis under drought stress in cowpea.

### 2.3. Unifoliate Leaf Chlorosis

**Figure 5:**
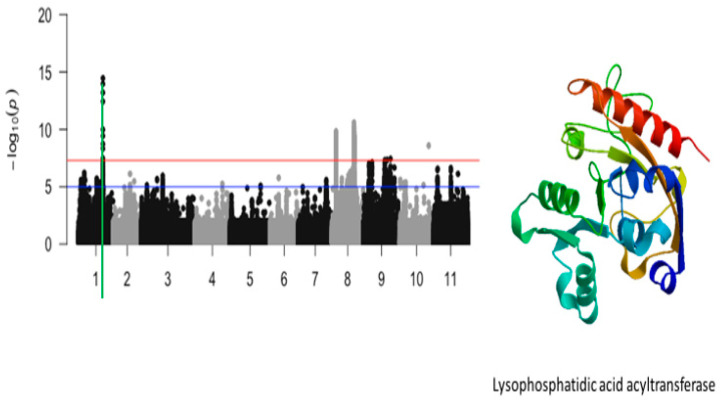
Manhattan plot for tolerance to unifoliate leaf chlorosis under drought in cowpea where the vertical green line defines a genomic region harboring a candidate gene encoding for lysophosphatidic acid acyltransferase.

**Figure 6:**
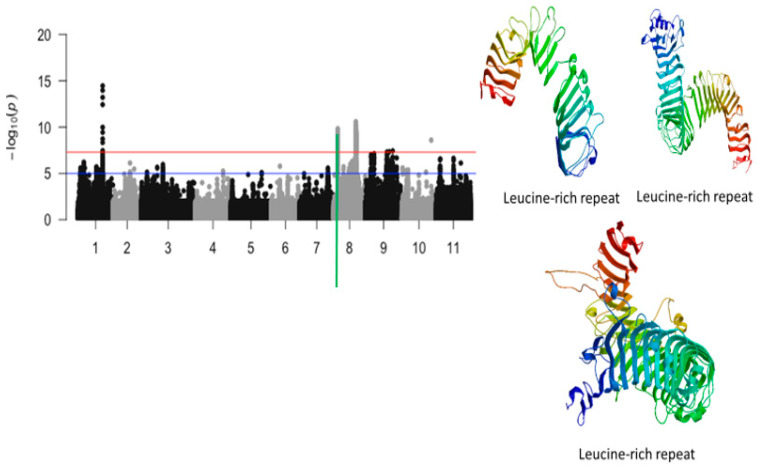
Manhattan plot for tolerance to unifoliate leaf chlorosis under drought in cowpea where the vertical green line defines a genomic region harboring candidate genes encoding for leucine-rich repeats.

**Figure 7:**
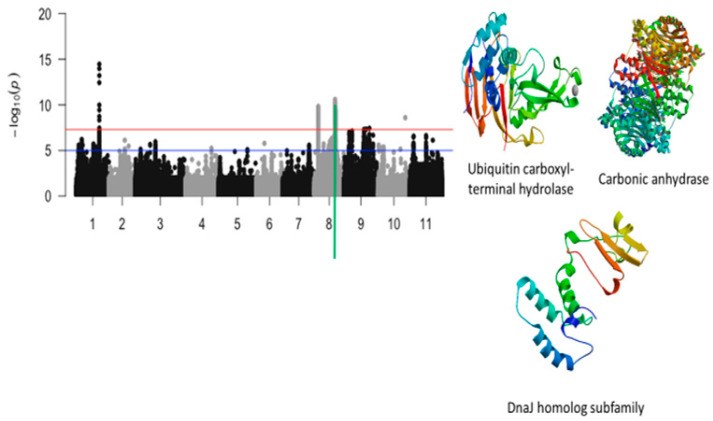
Manhattan plot for tolerance to unifoliate leaf chlorosis under drought in cowpea where the vertical green line defines a genomic region harboring candidate genes encoding for ubiquitin carboxyl terminal hydrolase, carbonic anhydrase, and DnaJ homolog subfamily.

**Figure 8:**
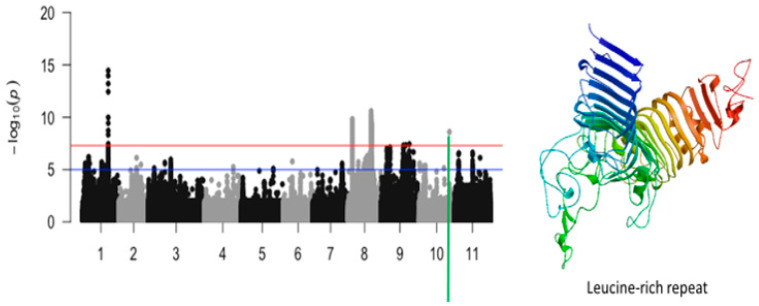
Manhattan plot for tolerance to unifoliate leaf chlorosis under drought in cowpea where the vertical green line defines a genomic region harboring a candidate gene encoding for leucine-rich repeat.

Tolerance to unifoliate leaf chlorosis has also been described as a mechanism for coping with water deficiency in cowpea. In this study, a total of 591 SNPs were found to be significantly associated with tolerance to unifoliate leaf chlorosis under drought stress. A total of 8, 582, and 1 significant SNPs were found on chromosomes 1, 8, and 10, respectively (Figure 4, Figure 5, Figure 6 and Figure 7). LOD (−log_10_(*p*-value)) values varied from 7.52 to 14.45 for the significant SNPs. The results indicated three significant loci associated with tolerance to unifoliate leaf chlorosis. These loci were mapped on chromosomes 1 and 8 (Figure 5, Figure 6, Figure 7 and Figure 8).

The significant locus that was identified on chromosome 1 was defined by a total of 8 SNPs. These SNPs were mapped on a 27 kb region of chromosome 1 (Figure 5). These SNPs were Vu01_29542433 (LOD = 9.98), Vu01_29544073 (LOD = 13.2), Vu01_29544191 (LOD = 14.45), Vu01_29544749 (LOD = 13.97), Vu01_29548480 (LOD = 12.43), Vu01_29549609 (LOD = 8.33), Vu01_29558145 (LOD = 8.72), and Vu01_29570238 (LOD = 9.43) (Table 1). A total of three annotated genes were found within this region. Of the three annotated genes, *Vigun01g119000.1* is the only one that has a functional annotation. *Vigun01g119000.1* encodes for lysophosphatidic acid acyltransferase (Figure 5). The significant SNP that was closest to this annotated gene was Vu01_29544191 (LOD = 14.45).

A 42 kb region of chromosome 8 contained a total of 65 SNPs that were significantly associated with tolerance to unifoliate leaf chlorosis under drought stress in cowpea (Figure 6). Of these SNPs, those with the highest LOD values were Vu08_4952393 (LOD = 9.83), Vu08_4946612 (LOD = 9.70), Vu08_4946618 (LOD = 9.70), Vu08_4945615 (LOD = 9.67), Vu08_4945627 (LOD = 9.61), Vu08_4946651 (LOD = 9.58), Vu08_4951347 (LOD = 9.51), Vu08_4951349 (LOD = 9.51), Vu08_4936939 (LOD = 9.40), Vu08_4946653 (LOD = 9.39), Vu08_4946682 (LOD = 9.39), Vu08_4946699 (LOD = 9.34), Vu08_4952509 (LOD = 9.34), Vu08_4952522 (LOD = 9.34), and Vu08_4952526 (LOD = 9.34). The significant locus defined by the 42 kb region of chromosome 8 harbored a cluster of three annotated genes encoding for a leucine-rich repeat (Table 1). The SNPs that were in the vicinity or within the structure of these annotated genes were Vu08_4931701 (LOD = 8.32), Vu08_4945627 (LOD = 9.61), and Vu08_4952526 (LOD = 10.59). The predicted tertiary structure of the protein derived from the three annotated genes was slightly different (Figure 6).

The third significant locus associated with tolerance to unifoliate leaf chlorosis was mapped on a 184 kb region of chromosome 8 (Figure 7). This region harbored a total of 517 significant SNPs. LOD (−log10(*p*-value)) values of the significant SNPs found in this region varied from 7.52 to 10.59. The SNPs with the highest LOD values were Vu08_26752606 (LOD = 10.59), Vu08_26852413 (LOD = 10.31), Vu08_26874709 (LOD = 10.27), Vu08_26898363 (LOD = 10.17), Vu08_26888097 (LOD = 10.11), Vu08_26877485 (LOD = 10.08), Vu08_26901689 (LOD = 10.04), Vu08_26878780 (LOD = 9.87), Vu08_26871649 (LOD = 9.83), Vu08_26871652 (LOD = 9.83), Vu08_26877438 (LOD = 9.78), Vu08_26874835 (LOD = 9.77), Vu08_26897604 (LOD = 9.77), and Vu08_26883655 (LOD = 9.75). The significant locus defined by the 184 kb region of chromosome 8 harbored seven annotated genes with six having functional annotations. The SNPs Vu08_26752606 (LOD = 10.59), Vu08_26868733 (LOD = 8.83), Vu08_26877485 (LOD = 10.08), and Vu08_26901689 (LOD = 10.04) were found in the vicinity or within the structure of *Vigun08g107800.1*, *Vigun08g107900.1*, *Vigun08g108100.1*, and *Vigun08g108400.1* encoding for Ubiquitin carboxyl-terminal hydrolase, AT-hook DNA-binding family protein, carbonic anhydrase, and DnaJ homolog subfamily, respectively. The predicted tertiary structure of these proteins is shown in Figure 7. One significant SNP located on chromosome 10 was also found to be associated with tolerance to unifoliate leaf chlorosis under drought stress in cowpea (Figure 8).

### 2.4. Plant Greenness Score

**Figure 9:**
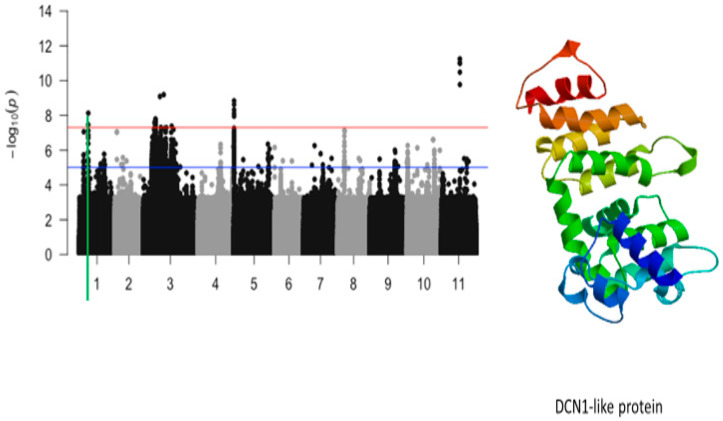
Manhattan plot for plant greenness score under drought stress in cowpea where the vertical green line defines a genomic region harboring a gene encoding for DNC1-like protein.

**Figure 10:**
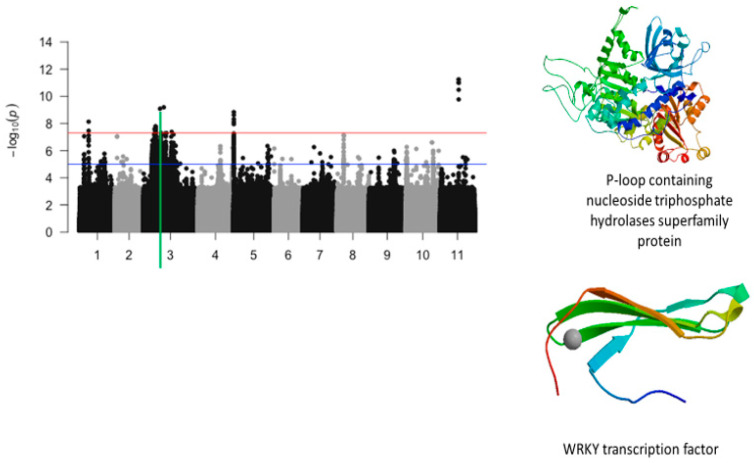
Manhattan plot for plant greenness score under drought stress in cowpea where the vertical green line defines a genomic region harboring candidate genes encoding for P-loop containing nucleoside triphosphate hydrolases superfamily protein and WRKY transcription factor.

**Figure 11:**
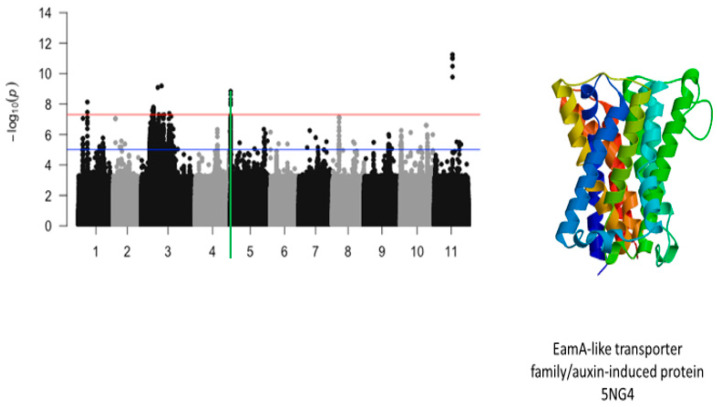
Manhattan plot for plant greenness score under drought stress in cowpea where the vertical green line defines a genomic region harboring a gene encoding for EamA-like transporter family/auxin-induced protein 5NG4.

Plant greenness score was recorded to assess the degree of wilting due to drought stress in this study. Unlike tolerance to trifoliate leaf chlorosis and unifoliate leaf chlorosis under drought conditions, a very few SNPs were identified to be associated with plant greenness score for the cowpea panel evaluated for drought tolerance at seedling stage. A total of 25 SNPs were identified and mapped on chromosomes 1, 3, 5, and 11 (Figure 9, Figure 10 and Figure 11). Chromosome 3 had the highest number of significant SNPs, whereas chromosome 1 had the lowest number of significant SNPs.

The significant SNPs associated with plant greenness score under drought stress consisted of Vu01_10616486 (LOD = 8.13), Vu03_13509429 (LOD = 7.58), Vu03_14725410 (LOD = 7.56), Vu03_14725434 (LOD = 7.78), Vu03_14725437 (LOD = 7.78), Vu03_14725438 (LOD = 7.78), Vu03_14725450 (LOD = 7.69), Vu03_14730296 (LOD = 7.59), Vu03_14730297 (LOD = 7.59), Vu03_14735109 (LOD = 7.58), Vu03_15042787 (LOD = 7.73), Vu03_20084616 (LOD = 9.09), Vu03_24643282 (LOD = 9.19), Vu05_540561 (LOD = 8.32), Vu05_541044 (LOD = 8.62), Vu05_541198 (LOD = 8.84), Vu05_541677 (LOD = 7.95), Vu05_544287 (LOD = 8.12), Vu11_22285237 (LOD = 11.00), Vu11_22285238 (LOD = 11.00), Vu11_22285251 (LOD = 11.00), Vu11_22285317 (LOD = 11.24), Vu11_22285318 (LOD = 11.24), Vu11_22285324 (LOD = 9.77), and Vu11_22285327 (LOD = 10.48). On chromosome 1, the SNP that was located in the vicinity of an annotated gene, *Vigun01g054900.1*, was Vu01_10616486 (LOD = 8.13). This gene encodes for DCN1-like protein. The predicted tertiary structure of this protein is shown in Figure 8. The genomic region harboring Vu01_10616486 contained also SNPs with relatively high LOD (−log_10_(*p*-value)) values as shown in Figure 9. However, these SNPs were just below the threshold that was chosen to declare significance in this study. The SNPs Vu03_13509429 (LOD = 7.58) and Vu03_14725438 (LOD = 7.78) were very close to the annotated genes *Vigun03g137600.1* and *Vigun03g144800.1*, respectively. The functional annotations of the proteins derived from these genes were the P-loop containing nucleoside triphosphate hydrolase superfamily protein and WRKY transcription factor, respectively. The predicted tertiary structure of these proteins is shown in Figure 10. Interestingly, the significant locus found at the beginning of chromosome 5 overlapped with the locus associated with tolerance to first trifoliate leaf chlorosis under drought stress (Figure 11). One significant SNP associated with plant greenness score and mapped on chromosome 5 was just located at 1 kb of another SNP having the highest LOD value for tolerance to trifoliate leaf chlorosis. These results indicate that this genomic result could control both the plant greenness score and tolerance to trifoliate leaf chlorosis under drought stress in cowpea. No annotated genes were found in the vicinity of the significant SNPs that were mapped on chromosome 11.

### 2.5. Protein Homologs

Protein homolog search was conducted for the candidate genes with functional annotations that are relevant to plant abiotic stress. The search was conducted within the genomes of legumes such as soybean, common bean, and Medicago. Only proteins with more than 90% with the query were considered. The search was also conducted within the cowpea genome to investigate potential gene duplication within the cowpea genome. For the candidate genes associated with trifoliate leaf chlorosis, the number of homologs significantly varied across species (Table 2). On average, the soybean genome has multiple copies of the same gene. The candidate genes *Vigun03g137500.1*, *Vigun03g135700.1*, and *Vigun04g110800.1* were unique within the cowpea genome. One or two copies of the candidate genes *Vigun05g006300.1*, *Vigun05g006500.1*, *Vigun03g136600.1*, and *Vigun04g110600.1* were identified within the cowpea genome (Table 2). The candidate genes *Vigun03g135800.1* and *Vigun03g135900.1* had more than four copies within the cowpea genome, seven copies within the soybean genome, five copies within the common bean genome, and four copies within the Medicago genome.

The results for tolerance to unifoliate leaf chlorosis were interesting in a way because most of candidate genes were unique in the cowpea genome (Table 2). Candidate genes consisting of *Vigun08g046400.1*, *Vigun08g107800.1*, *Vigun08g108100.1*, *Vigun08g108400.1*, and *Vigun10g137100.1* were unique within the cowpea genome. In addition, no copy of *Vigun10g137100.1* was found within the genome of soybean, common bean, and Medicago. Overall, gene duplication of the candidate genes associated with tolerance to unifoliate leaf chlorosis seemed to be more significant within the common bean genome. The results for plant greenness score were also similar to that of tolerance to unifoliate leaf chlorosis under drought stress. In fact, *Vigun01g054900.1*, *Vigun03g137600.1*, and *Vigun03g144800.1* were unique within the cowpea genome and only one copy was found for *Vigun05g006300.1*. No copies of *Vigun03g137600.1* were identified within the genome of common bean and Medicago. One copy of this gene was found within the soybean genome.

### 2.6. Overlapping SNPs and Functional Annotations

**Figure 12:**
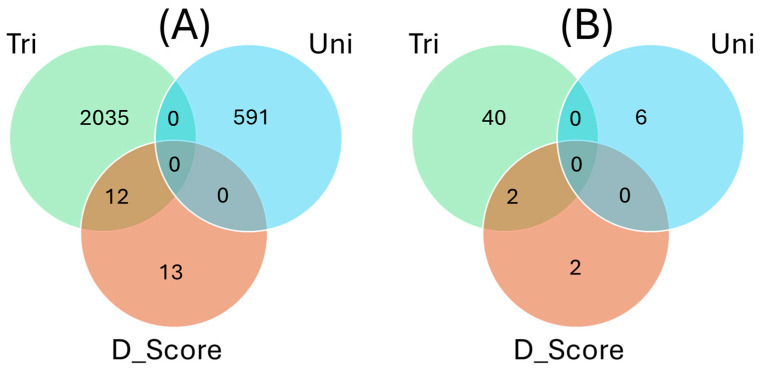
(**A**) Venn diagram showing the number of overlapping significant SNPs associated with tolerance to trifoliate leaf chlorosis (Tri), unifoliate leaf chlorosis (Uni), and plant greenness score (D_Score) under drought stress in cowpea. (**B**) Venn diagram showing the number of unique functional annotations for candidate genes associated with tolerance to trifoliate leaf chlorosis (Tri), unifoliate leaf chlorosis (Uni), and plant greenness score (D_Score) under drought stress in cowpea. Venn diagrams were established using http://jvenn.toulouse.inra.fr/app/example.html (Accessed on 12 September 2023).

The number of overlapping SNPs between traits was visualized using a Venn diagram (Figure 12). The number of SNPs associated with tolerance to trifoliate leaf chlorosis, tolerance to unifoliate leaf chlorosis, and plant greenness score was 1047, 591, and 25, respectively. On the Venn diagram, SNPs associated with trifoliate leaf chlorosis, unifoliate leaf chlorosis, and plant greenness score were represented by solid green, blue, and pink circles, respectively (Figure 12). No overlapping SNPs were found between the three traits investigated for drought stress. However, a total of 12 SNPs overlapped between tolerance to trifoliate leaf chlorosis and plant greenness score in cowpea. No common SNPs were found between tolerance to unifoliate leaf chlorosis and plant greenness score. In addition, no overlapping SNPs were identified between tolerance to trifoliate leaf chlorosis and tolerance to unifoliate leaf chlorosis under drought stress in cowpea, indicating that these two traits could have independent genetic mechanism.

## 3. Discussion

To the best of our knowledge, this is the first report on GWAS for drought tolerance in cowpea using a whole genome resequencing data. A total of 14,465,516 SNPs were obtained from whole genome resequencing, of which, 5,884,299 SNPs satisfied the filtering criteria and were further processed for GWAS. To date, this could be the largest amount of SNPs data that was used to conduct GWAS in cowpea. We have identified strong GWAS peaks that were associated with tolerance to trifoliate leaf chlorosis, tolerance to unifoliate leaf chlorosis, and plant greenness under drought stress in cowpea at seedling stage. In addition to the individual GWAS peaks, a large number of significant SNPs were also identified and scattered across the cowpea genome, which could support earlier reports suggesting that drought tolerance is a complex mechanism.

In this study, a total of 1047, 591, and 25 SNPs were identified to be associated with tolerance to trifoliate leaf chlorosis, tolerance to unifoliate leaf chlorosis, and plant greenness score under drought stress, respectively. Interestingly no overlapping SNPs were found between the three traits. No common SNPs were identified between tolerance to trifoliate leaf chlorosis and tolerance to unifoliate leaf chlorosis. This could explain previous studies stating that there are two types of drought tolerance in cowpea and the mechanisms underlying these two types were independent [34]. The type I drought-tolerant cowpea genotypes can delay senescence in both trifoliate and unifoliate leaves. However, the type II ones kept the trifoliate leaf green, but they were more susceptible to unifoliate leaf chlorosis [31]. The strong GWAS peak on chromosome 5, which was associated with tolerance to trifoliate leaf chlorosis, was included in a significant drought-tolerant QTL region reported by [12]. A QTL mapping study for drought tolerance in cowpea has been conducted using the population derived from a cross between IT93K503-1 (drought-tolerant) and CB46 (drought-susceptible). Visual rating on leaf senescence under drought conditions for that population was conducted, and a QTL was identified within a 15-cM distance [12]. Therefore, our results refined this QTL region.

Candidate genes involved in hormone biosynthesis pathways and membrane lipid degradation were also identified in this study. These genes were previously described as being directly involved in drought stress in cowpea. A total of 24 cDNA clones pertaining to dehydration-induced genes were isolated from a highly drought-tolerant cowpea cultivar (IT84S-2246-4) [35]. Of the 24 cDNAs, 9 were induced by water-deficit conditions. Five of them were characterized and known as cowpea clones responsive to dehydration (CRPD) genes (*CPRD8*, *CPRD12*, *CPRD14*, *CPRD22*, and *CPRD46*). Another CPRD gene (*CPRD86*) was studied later [36]. Two additional drought-tolerant genes, *VuNCED1* and *VuABA1*, were described and isolated from the aforementioned cultivar [36]. Investigations showed that *VuNCED1* encodes a 9-cis-epoxycarotenoid dioxygenase catalyzing a key step in the abscisic acid (ABA) biosynthesis [35,36]. ABA is involved in abiotic stress signaling, which triggers the plant defense system to stimulate responses to water-deficit conditions. *VuABA1* was demonstrated to encode a zeaxanthin epoxidase involved in another significant pathway for abscisic acid (ABA) biosynthesis [35]. Therefore, *VuABA1* is critical in enabling ABA biosynthesis. A study described the *VuPLD1* gene encoding a phospholipase D, which is stimulated by drought stress [37]. Indeed it is widely recognized that lipid metabolism is triggered upon degradation of membrane lipids under drought conditions [38]. The results revealed a highly expressed *VuPLD1* in a cowpea drought-susceptible cultivar to which drought stress was imposed; meanwhile, its expression was unchanged in a drought-tolerant one [37].

A previous research study also described two cowpea cDNAs, *VuPAP-α* and *VuPAP-β*, encoding putative phosphatide phosphatases (PAPs) [39]. Previous research has shown that PAPs were significantly involved in the pathway related to membrane lipid degradation for plants under abiotic stresses or senescence [40]. The expression of *VuPAP-α* was stimulated for cowpea genotypes submitted to rehydration under a certain period of drought [39]. However, the expression of *VuPAP-β* was increased in air-desiccated leaves from which a cowpea *VuPAT1* (putative patatin-like) gene encoding for galactolipid acyl hydrolase was isolated and characterized, whose expression was increased in a cowpea cultivar susceptible to drought [41]. Galactolipids, components of chloroplast membrane, were hydrolyzed in cowpea genotypes under drought stress [42]. The cowpea *VuC1* gene encoding for cowpea cystatin is a leaf protease inhibitor regulating protein degradation and prevents leaf cells from oxidative damage under drought conditions [3,43]. Two cowpea genes, *dtGR* and *cGR*, encode for dual-targeted glutathione reductase and cytosolic glutathione reductase, respectively [10]. These are key enzymes involved in the detoxification of antioxidant metabolites under progressive drought conditions.

Further cowpea antioxidant genes related to drought stress were isolated and characterized [44]. These genes encode for cytosolic ascorbate peroxidase (*VucAPX*), peroxisomal ascorbate peroxidase (*VupAPX*), stromatic ascorbate peroxidase (*VusAPX*), and thylakoidal ascorbate peroxidase (*VutAPX*). These enzymes are involved in detoxifying antioxidant species under drought stress in cowpea [10]. Two additional abiotic-stress cowpea-related genes, *GST* (glutathione-S-transferase) and *PR-1* (pathogenesis-related-protein-1), were previously described [45]. The research has highlighted the effects of drought and heat on cowpea nodules [46]. The results have revealed that the genes *VuNSR4*, *VuNSR10*, *VuNSR44*, *VuNSR47*, and *VuNSR49*, encoding for digalactosildiacylglycerol synthase 1, kinase protein calcium-dependent, CPRD12, CPRD8, and CPRD65, respectively, played a significant role in protecting cowpea nodules from drought and heat stresses. In addition to being regulated by proteins translated from genes, cowpea drought tolerance is also controlled by the effects of microRNAs (miRNAs). A total of 44 miRNAs were reported to be associated to drought stress in cowpea [47,48]. The number of genes that are involved in drought stress tolerance in cowpea suggested the complexity of this trait [3]. However, a study reported that a major gene could control drought tolerance in cowpea [49]. In fact, crosses between TX2028-1-3-1 (drought-tolerant) and TVu-7778 (drought-susceptible), and TX2028-1-3-1 (drought-tolerant) and CB 46 (drought-susceptible) showed a segregation ratio of 3:1 for unifoliate stay-green trait in F_2_ progenies. Therefore, further investigations are required to unravel more possible mechanisms of drought tolerance at the genetic level in cowpea.

This study has provided molecular markers associated with drought tolerance at the seedling stage in cowpea. However, the significant SNP markers have not yet been validated. Therefore, an additional study should be conducted to validate the SNP markers so that they can be reliably used for Marker-Assisted Selection (MAS). In addition, the results from this study contribute towards understanding the genetic architecture of drought tolerance in cowpea. The functional annotations of the annotated genes found within or in the vicinity of the location of the significant SNPs have provided substantial hints on potential drought tolerance mechanism. These candidate genes will be validated in further projects. The SNPs associated with drought tolerance that were identified in this study can be used to develop Kompetitive Allele Specific Polymerase Chain Reaction (KASP) markers to rapidly select for new more drought-tolerant cowpea lines. This will further decrease the costs associated with drought tolerance phenotyping. Despite the large amount of data generated in this study, one major limitation relates to the fact that the screening was conducted at seedling stage and under greenhouse conditions. To date, we do not have enough information whether these results can be replicated at reproductive stage and under field conditions. Further investigations are required to address this constraint.

## 4. Materials and Methods

### 4.1. Plant Materials and Phenotyping

Seedling drought tolerance was evaluated using a panel of 331 cowpea genotypes (Table S1) consisting of 36 breeding lines from the University of Arkansas, Fayetteville, 8 from the University of California, Riverside that were the founders of the first multiparent advanced generation intercross (MAGIC) population [50], and 287 Plant Introductions (PIs) from the U.S. Department of Agriculture (USDA) Germplasm Resources Information Network (GRIN) cowpea accessions. The PIs were obtained from the USDA Plant Genetic Resources Conservation Unit at Griffin, GA, USA. The cowpea genotypes were from more than 32 countries. Seed increase was conducted in the summer of 2018 at the Arkansas Agricultural Experiment Station of the University of Arkansas, Fayetteville. One plant from each genotype was harvested. Cleaned and carefully sorted seeds from each plant were used for the experiments.

Cowpea drought tolerance study was performed in a greenhouse at the University of Arkansas, Fayetteville, AR. Screening methodology was previously described [51]. Sterilite polypropylene boxes (Sterilite Corporation, Townsend, MA, USA) were used for drought phenotyping. Boxes were 88.6 cm long, 42.2 cm wide, and 15.6 cm high. Sunshine^®^ Mix #1 Natural & Organic (Agawan, MA, USA) was used as a soil medium. Boxes were watered with 12 L of tap water two days before sowing so that field capacity was attained at planting time [49]. The greenhouse average day/night temperatures were 26 °C/21 °C. The daylight length was about 14 h during the experiments.

A total of 10 rows were designed at each 7.5 cm through the box length. For each genotype, two cowpea seeds were sown in a 2 cm diameter hole across each row containing a total of 12 seeds. Cowpea plants were thinned to one plant per hole upon plant establishment so that six plants remain within each row. A solution of 150 mL Miracle-Gro fertilizers (Scotts Miracle-Gro, Detroit, MI, USA) were applied to each row at one week after plant emergence. Fertilizer solution was obtained by dissolving one tablespoon of Miracle-Gro into one gallon of tap water. Each row was irrigated with 150 mL tap water each three days and until the first trifoliate leaf was fully expanded. Plants were watered until the first trifoliate leaf was fully expanded and watering was stopped after this time in the drought-stressed box. Irrigation was still conducted in the well-watered box. Drought-tolerant genotypes included PI293469, PI349674, and PI293568, and drought-susceptible check was PI255774 [51]. The experiments were conducted using 3 runs and each run was considered as a blocking variable. The experimental unit corresponded to each row within boxes. Soil moisture was assessed using an HH2 Moisture Meter (Delta-T Devices, Cambridge, UK) every 3 days. Data measurements were previously described [51]. The phenotypic data included trifoliate leaf chlorosis, unifoliate leaf chlorosis, and plant greenness score. Tolerance to trifoliate leaf chlorosis is indicative of a type I drought stress tolerance in cowpea, whereas tolerance to unifoliate leaf chlorosis is a type II drought stress tolerance. Plant greenness score is a combination of both types of drought tolerance.

### 4.2. Genotyping

#### 4.2.1. DNA Extraction, Library Preparation, and Whole-Genome Resequencing

DNA was extracted from a single plant of each line using the CTAB (hexadecyltrimethyl ammonium bromide) protocol [52]. Harvested leaf tissues were ground in Mixer Mill MM 400^®^ (Haan, Germany). DNA buffer was added to samples that were centrifuged at 13,000 rpm for 10 min. Proteins were denatured by adding 1 mL of chloroform-isoamyl alcohol (24:1) to each sample. DNA was precipitated by adding 1 mL of isopropanol to each sample. Samples were stored at −20 °C overnight. DNA pellets were washed by 70% and 90% ethanol. After ethanol washing, samples were air-dried. RNA was removed by adding 3 µL of RNAse to each sample. DNA was stored in a solution of 200 µL of 0.1X TE. The amount of DNA within each sample was quantified using a NanoDrop 200c spectrophotometer (Thermo Scientific, Wilmington, DE, USA). DNA was quality-checked on a 1%-agarose gel with ethidium bromide stain.

DNA sequencing was performed by Novogene (http://en.novogene.com/; Accessed on 12 September 2023). Cleavage of DNA was performed using Covaris S2^®^ (Covaris, Inc., Woburn, MA, USA). This generated a set of approximately 350 bp DNA fragments. DNA library consisted of sheared DNA fragments and NEBNext DNA Library Prep Reagent Set for Illumina (BioLabs, Inc., Ipswich, MA, USA). DNA fragments were end-repaired. Poly-A tails were added to each fragment. Fragmented DNA was purified and subjected to in situ PCR amplification as previously described [53]. Genomic DNA sequencing was achieved using Illumina HiSeq X Ten Series (http://www.illumina.com/systems/hiseq-x-sequencing-system/system.html; Accessed on 12 September 2023) with an average of 10X coverage. This study involved a total of more than 1.88 Tb of genomic information sequence.

#### 4.2.2. SNP Calling, Mapping, and Filtering

Short-reads were aligned to the cowpea reference genome *Vigna unguiculata* v1.1 [54]. Alignments were performed using SOAPaligner/soap2 (https://gaow.github.io/genetic-analysis-software/s/soap/; Accessed on 12 September 2023). Preliminary SNP calling was achieved using SOAPsnp v 1.05 [55]. Accessions having more than 20% missing SNP information were removed. Triallelic SNPs and those with more than 20% missing data were also not considered for GWAS. SNPs with more than 20% heterozygous calls were discarded from the analysis. The minor allele frequency (MAF) threshold was 5%. GWAS was conducted using filtered SNPs.

### 4.3. Population Structure and Genetic Diversity Analysis

Population structure analysis was conducted using STRUCTURE 2.3.3 (https://web.stanford.edu/group/pritchardlab/structure_software/release_versions/v2.3.3/html/structure.html; Accessed on 12 September 2023). An admixture model and a correlated allele frequency model were used. The burn-in period Markov chain Monte Carlo (MCMC) length was 20,000, and the number of MCMC iterations was set to 20,000. Genetic diversity was established using MEGA 7 (https://www.megasoftware.net/; Accessed on 12 September 2023). The grouping from the population structure analysis was used as an additional parameter to establish genetic diversity. The color coding on the genetic diversity was consistent with the grouping (Q matrix) from the population structure analysis. If an accession was grouped into a class, a color and shape corresponding to that class was designed and used to format the genetic diversity tree. Population structure reduced false positive discovery [31,32].

### 4.4. Genome Wide Association Study (GWAS)

GWAS was conducted using a Bayesian information and linkage disequilibrium iteratively nested keyway (BLINK) model [56]. BLINK has been demonstrated to effectively reduce false positive discovery [56]. SNP was declared to be significant when above the FDR-adjusted threshold and computed in R (*p* < 3 × 10^−8^). BLINK model was derived from the fixed and random model circulating probability unification (FarmCPU) model. FarmCPU assumed markers being evenly distributed across the genome, which could be easily violated. Instead, BLINK used the LD information to relax this assumption. In addition, the heavy computational-related issue due to the random effect model (REM) was replaced by a second, fixed model (FEM) in BLINK. The two FEM models in BLINK are described below.

$$FEM\;(1)\;y_{i}=M_{i1}b_{1}+M_{i2}b_{2}+\ldots +M_{ik}b_{k}+M_{ij}d_{j}+e_{i}\;$$

$$FEM\;(2)\;y_{i}=M_{i1}b_{1}+M_{i2}b_{2}+\ldots +M_{ij}d_{j}+e_{i}$$

with *y*_i_ being the vector phenotype; *M_i_*_1_, *M_i_*_2_*b*_2_, …, *M_ik_* the genotypes of *k* pseudo QTNs having effects *b*_1_, *b*_2_, …, *b*_k_, respectively; *M_ij_* corresponds to the *j*th genetic marker of the *i*th sample; and *e_i_* represents the residual having a distribution with mean zero and a variance σ^2^_e_. Overlapping SNP markers between different traits were visualized using a Venn diagram that was designed using the online software program accessible at http://jvenn.toulouse.inra.fr/app/example.html (Accessed on 12 September 2023).

### 4.5. Candidate Gene Search and Synteny Analysis

Significant SNPs was used to investigated gene search within 10 bk genomic region flanking a SNP using Phytozome v.13 (https://phytozome.jgi.doe.gov/pz/portal.html; accessed on 12 September 2023). Annotated genes having functional annotations relevant to plant physiology and/or tolerance to abiotic stress were considered. Functional annotations were also obtained from Phytozome v. 13. Coding sequences of the candidate gene of interest were extracted and used as input for BLASTx (https://blast.ncbi.nlm.nih.gov/Blast.cgi; Accessed on 12 September 2023) to identify the amino acid sequence used to conduct protein homolog search in other legumes such as soybean, common bean, and *Medicago truncatula* Gaertn. Only hits with a similarity greater than 90% were considered. The tertiary structure of the polypeptide/protein that was derived from the amino acid sequence was predicted using SWISS-MODEL (https://swissmodel.expasy.org/; Accessed on 12 September 2023).

## 5. Conclusions

The whole genome resequencing provided a total of 14,465,516 SNPs. GWAS was conducted using a total of 5,884,299 filtered SNPs. A total of 1047, 591, and 25 SNPs were found to be associated with tolerance to trifoliate leaf chlorosis, tolerance to unifoliate leaf chlorosis, and plant greenness score under drought stress, respectively. A strong candidate locus was mapped on a 210 kb of chromosome 5 and associated with tolerance to trifoliate leaf chlorosis. This region harbored hormone-induced genes. A strong GWAS peak was also identified for tolerance to unifoliate leaf chlorosis. A total of 12 overlapping SNPs were found for tolerance to trifoliate leaf chlorosis and plant greenness score under the drought score in cowpea. These results could be used in cowpea breeding through marker-assisted selection (MAS). Molecular markers can be designed using the SNPs reported in this study. The use of these molecular markers can speed up the selection of drought tolerance trait in cowpea. The findings can be also expanded to other crop species by finding syntenic regions associated with the drought tolerance trait. Future projects will include validating these markers in different environments and growth stages. To the best of our knowledge, this is the first report on cowpea GWAS for drought tolerance using a whole genome resequencing data.

## Figures and Tables

**Table 1:** List of significant SNPs close to candidate genes and associated with tolerance to trifoliate leaf chlorosis and unifoliate leaf chlorosis, and plant greenness under drought tolerance in cowpea. SNP, base pair (BP), chromosome (CHR), Pval, and logarithm of odds (LOD) refers to SNP_ID, chromosome number, physical location (in bp), *p*-value, and −log10 of *p*-value (LOD), respectively. Gene_ID and functional annotations were obtained from Pythozome v.13.

Traits	SNP	BP	CHR	Pval	LOD	Gene_ID	Functional_Annotation
Trifoliate leaf chlorosis	Vu01_10616309	10616309	1	6.54 × 10^−9^	8.18	*Vign01g055000.1*	Mn plant transporter
	Vu01_25892353	25892353	1	3.74 × 10^−9^	8.43	*Vign01g09400.1*	NADH-cytochrome b5 reductase
	Vu02_24537084	24537084	2	1.12 × 10^−8^	7.95	*Vign02090500.1*	Retrotransposon
	Vu03_10803196	10803196	3	2.98 × 10^−8^	7.53	*Vign03g116500.1*	PB1 domain containing protein
	Vu03_12666912	12666912	3	2.04 × 10^−8^	7.69	*Vigun03g130400.1*	Protein phosphatase 2C
	Vu03_12897768	12897768	3	1.55 × 10^−8^	7.81	*Vign03g132300.1*	Pumilio-family RNA binding repeat
	Vu03_13212508	13212508	3	2.99 × 10^−8^	7.52	*Vign03g135100.1*	ATP-dependent DNA helicase 2 subunit 2
	Vu03_13274473	13274473	3	1.89 × 10^−8^	7.72	*Vign03g135400.1*	RNA methylase-related
	Vu03_13295491	13295491	3	5.40 × 10^−10^	9.27	*Vign03g135700.1*	Vacuolar iron transporter
	Vu03_13297714	13297714	3	5.68 × 10^−12^	11.25	*Vign03g135800.1*	Vacuolar iron transporter
	Vu03_13302250	13302250	3	1.84 × 10^−11^	10.74	*Vign03g135900.1*	Vacuolar iron transporter
	Vu03_13352276	13352276	3	1.16 × 10^−9^	8.93	*Vigun03g136300.1*	EamA-like transporter family/Glucose-6-phosphate/phosphate and phosphoenolpyruvate/phosphate antiporter
	Vu03_13361294	13361294	3	5.04 × 10^−10^	9.30	*Vigun03g136400.1*	EamA-like transporter family/Glucose-6-phosphate/phosphate and phosphoenolpyruvate/phosphate antiporter
	Vu03_13376628	13376628	3	1.96 × 10^−10^	9.71	*Vigun03g136500.1*	EamA-like transporter family/Glucose-6-phosphate/phosphate and phosphoenolpyruvate/phosphate antiporter
	Vu03_13382599	13382599	3	2.55 × 10^−10^	9.59	*Vigun03g136600.1*	EamA-like transporter family/auxin-induced protein 5NG4
	Vu03_13509429	13509429	3	5.66 × 10^−11^	10.25	*Vigun03g137500.1*	ABA responsive element binding factor
	Vu03_14318570	14318570	3	9.08 × 10^−10^	9.04	*Vigun03g142100.1*	Tetrahydroberberine oxidase
	Vu03_14743049	14743049	3	5.21 × 10^−9^	8.28	*Vigun03g144800.1*	WRKY TRANSCRIPTION FACTOR 28-RELATED
	Vu03_14763808	14763808	3	2.88 × 10^−8^	7.54	*Vigun03g144900.1*	O-acetyltransferase family protein
	Vu03_14806565	14806565	3	2.32 × 10^−9^	8.63	*Vigun03g145200.1*	Starch synthase
	Vu03_14815803	14815803	3	1.63 × 10^−9^	8.79	*Vigun03g145400.1*	Chlorophyll a/b binding protein
	Vu03_15222570	15222570	3	2.69 × 10^−8^	7.57	*Vigun03g148300.1*	3-oxoacyl-synthase
	Vu03_36340055	36340055	3	2.93 × 10^−9^	8.53	*Vigun03g218100.1*	ABC-2 type transporter family protein
	Vu03_58980712	58980712	3	3.74 × 10^−9^	8.43	*Vigun03g384600.1*	H+-transporting ATPase
	Vu04_26966450	26966450	4	4.24 × 10^−9^	8.37	*Vigun04g109300.1*	CemA-like proton extrusion protein-related
	Vu04_27157237	27157237	4	6.15 × 10^−9^	8.21	*Vigun04g109500.1*	Protein FLOWERING LOCUS T (FT)
	Vu04_27241963	27241963	4	4.99 × 10^−9^	8.30	*Vigun04g109600.1*	TatD DNase family protein
	Vu04_27298716	27298716	4	5.99 × 10^−9^	8.22	*Vigun04g109700.1*	Aquaporin-like superfamily protein
	Vu04_27342140	27342140	4	2.73 × 10^−9^	8.56	*Vigun04g109800.1*	Nucleoside-triphosphatase
	Vu04_27505387	27505387	4	3.11 × 10^−9^	8.51	*Vigun04g110000.1*	rRNA-processing protein FCF
	Vu04_27528973	27528973	4	7.92 × 10^−9^	8.10	*Vigun04g110100.1*	Ubiquinone (electron-transporting coenzyme) biosynthesis protein
	Vu04_27714135	27714135	4	1.91 × 10^−9^	8.72	*Vigun04g110600.1*	No apical meristem (NAM) protein
	Vu04_27716250	27716250	4	4.48 × 10^−9^	8.35	*Vigun04g110700.1*	Tryptophan/tyrosine permease family
	Vu04_27778870	27778870	4	2.16 × 10^−8^	7.67	*Vigun04g110800.1*	Myb family transcription factor-related
	Vu04_27786623	27786623	4	8.27 × 10^−10^	9.08	*Vigun04g110900.1*	Pyruvate kinase
	Vu04_27797389	27797389	4	4.32 × 10^−9^	8.37	*Vigun04g111000.1*	Zinc finger protein
	Vu04_27830859	27830859	4	1.57 × 10^−8^	7.81	*Vigun04g111100.1*	CCR4-NOT transcription factor
	Vu04_27913211	27913211	4	1.59 × 10^−8^	7.80	*Vigun04g111200.1*	Glucan endo-1,3-beta-D-glucosidase
	Vu04_27913980	27913980	4	8.65 × 10^−9^	8.06	*Vigun04g111300.1*	GATA zinc finger
	Vu04_41785910	41785910	4	3.13 × 10^−9^	8.50	*Vigun04g193600.1*	Protein kinase superfamily
	Vu04_41800041	41800041	4	2.14 × 10^−8^	7.67	*Vigun04g193700.1*	NAD-dependent epimerase/dehydratase
	Vu04_41826262	41826262	4	7.69 × 10^−9^	8.11	*Vigun04g193800.1*	Translation initiation factor 3 subunit G
	Vu04_41832927	41832927	4	8.14 × 10^−9^	8.09	*Vigun04g194000.1*	Universal stress protein family
	Vu05_540561	540561	5	5.09 × 10^−21^	20.29	*Vigun05g006300.1*	EamA-like transporter family/auxin-induced protein 5NG4
	Vu05_560665	560665	5	5.63 × 10^−15^	14.25	*Vigun05g006500.1*	Neoxanthin synthase/abscisic acid biosynthesis
	Vu07_23856082	23856082	7	5.11 × 10^−10^	9.29	*Vigun07g129600.1*	Protein tyrosine kinase
	Vu07_24143183	24143183	7	7.91 × 10^−9^	8.10	*Vigun07g131800.1*	Protoporphyrinogen oxidase/chloroplast precursor
	Vu08_37171764	37171764	8	2.43 × 10^−8^	7.61	*Vigun08g208700.1*	PPR repeat
Unifoliate leaf chlorosis	Vu01_29544191	29544191	1	3.51 × 10^−15^	14.45	*Vigun01g119000.1*	Lysophosphatidic acid acyltransferase
	Vu08_4931701	4931701	8	4.83 × 10^−9^	8.32	*Vigun08g046200.1*	Leucine-rich repeat
	Vu08_4945627	4945627	8	2.46 × 10^−10^	9.61	*Vigun08g046400.1*	Leucine-rich repeat
	Vu08_4952526	4952526	8	4.61 × 10^−10^	9.34	*Vigun08g046500.1*	Leucine-rich repeat
	Vu08_26752606	26752606	8	2.58 × 10^−11^	10.59	*Vigun08g107800.1*	Ubiquitin carboxyl-terminal hydrolase
	Vu08_26868733	26868733	8	1.49 × 10^−9^	8.83	*Vigun08g107900.1*	AT-hook DNA-binding family protein
	Vu08_26877485	26877485	8	8.31 × 10^−11^	10.08	*Vigun08g108100.1*	Carbonic anhydrase
	Vu08_26901689	26901689	8	9.11 × 10^−11^	10.04	*Vigun08g108400.1*	DnaJ homolog subfamily
	Vu10_35348050	35348050	10	2.58 × 10^−9^	8.59	*Vigun10g137100.1*	Leucine-rich repeat
Plant greenness	Vu01_10616486	10616486	1	7.42 × 10^−9^	8.13	*Vigun01g054900.1*	DCN1-like protein
	Vu03_13509429	13509429	3	2.63 × 10^−8^	7.58	*Vigun03g137600.1*	P-loop containing nucleoside triphosphate hydrolase superfamily protein
	Vu03_14725438	14725438	3	1.65 × 10^−8^	7.78	*Vigun03g144800.1*	WRKY transcription factor
	Vu05_541044	541044	5	2.40 × 10^−9^	8.62	*Vigun05g006300.1*	EamA-like transporter family/auxin-induced protein 5NG4

**Table 2:** List of candidate genes having functional annotations that are relevant to plant abiotic stress. Protein homologs from each translated transcript were searched in the cowpea (Vun), soybean (Gma), common bean (Pvu), and Medicago truncatula (Mtr) genomes. The number of protein homologs with similarity > 90% to that one from cowpea is reported.

Traits	Gene_ID	Functional_Annotations	Vun	Gma	Pvu	Mtr
Trifoliate leaf chlorosis	*Vigun05g006300.1*	EamA-like transporter family/auxin-induced protein 5NG4	1	3	4	1
	*Vigun05g006500.1*	Neoxanthin synthase/abscisic acid biosynthesis	1	3	3	3
	*Vigun03g136600.1*	EamA-like transporter family/auxin-induced protein 5NG4	2	5	3	3
	*Vigun03g137500.1*	ABA responsive element binding factor	0	1	1	0
	*Vigun03g135700.1*	Vacuolar iron transporter	0	1	1	0
	*Vigun03g135800.1*	Vacuolar iron transporter	4	9	4	8
	*Vigun03g135900.1*	Vacuolar iron transporter	6	7	7	4
	*Vigun04g110600.1*	No apical meristem (NAM) protein	1	4	2	0
	*Vigun04g110800.1*	Myb family transcription factor-related	0	2	1	1
Uniifoliate leaf chlorosis	*Vigun01g119000.1*	Lysophosphatidic acid acyltransferase	1	4	2	2
	*Vigun08g046200.1*	Leucine-rich repeat	2	2	6	0
	*Vigun08g046400.1*	Leucine-rich repeat	0	1	4	0
	*Vigun08g046500.1*	Leucine-rich repeat	1	2	4	0
	*Vigun08g107800.1*	Ubiquitin carboxyl-terminal hydrolase	0	0	1	0
	*Vigun08g108100.1*	Carbonic anhydrase/Carbonate dehydratase	0	1	1	1
	*Vigun08g108400.1*	DnaJ homolog subfamily	0	2	1	1
	*Vigun10g137100.1*	Leucine-rich repeat	0	0	0	0
Plant greenness	*Vigun01g054900.1*	DCN1-like protein	0	2	1	1
	*Vigun03g137600.1*	P-loop containing nucleoside triphosphate hydrolase superfamily protein	0	1	0	0
	*Vigun03g144800.1*	WRKY transcription factor	0	2	1	0
	*Vigun05g006300.1*	EamA-like transporter family/auxin-induced protein 5NG4	1	3	3	1

## Data Availability

Data are within the manuscript.

## Supplementary Materials

The following supporting information can be downloaded at: https://www.mdpi.com/article/10.3390/ijms26125478/s1.
